# Supplementary figures and images for: Comparative analysis of gene expression identifies distinct molecular signatures of bone marrow- and periosteal-skeletal stem/progenitor cells
Source: PLoS One. 2018 Jan 17;13(1):e0190909. doi: 10.1371/journal.pone.0190909 (PMC5771600; doi:10.1371/journal.pone.0190909)

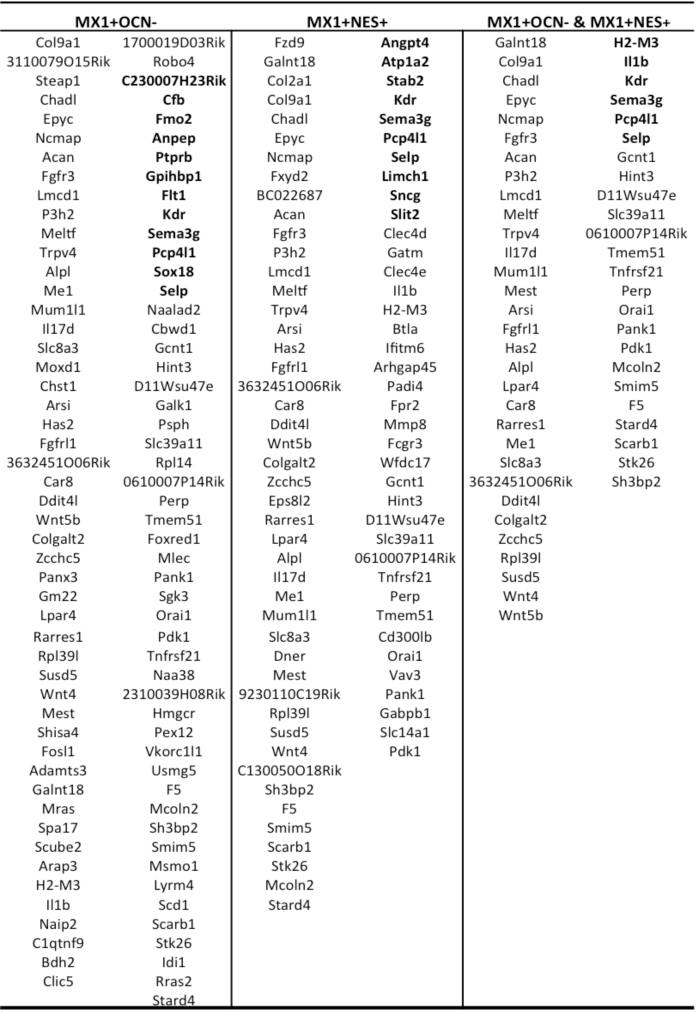

Supplement: S1 Table — SSCs are compared with both CD45+ cells and Osx+ cells (p < 0.05). (TIF) [file pone.0190909.s001.tif]

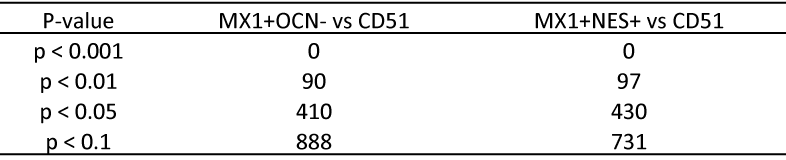

Supplement: S2 Table — (TIF) [file pone.0190909.s002.tif]
